# Visible Light Communication System Using an Organic Bulk Heterojunction Photodetector

**DOI:** 10.3390/s130912266

**Published:** 2013-09-12

**Authors:** Belén Arredondo, Beatriz Romero, José Manuel Sánchez Pena, Agustín Fernández-Pacheco, Eduardo Alonso, Ricardo Vergaz, Cristina de Dios

**Affiliations:** 1 Electronic Technology Department, University Rey Juan Carlos, Calle Tulipán s/n, Móstoles 28933, Madrid, Spain; E-Mail: beatriz.romero@urjc.es; 2 Electronic Technology Department, University Carlos III de Madrid, Avda. de la Universidad, 30, Leganés 28911, Madrid, Spain; E-Mails: jmpena@ing.uc3m.es (J.M.S.P.); afroman@ing.uc3m.es (A.F.-P.); eduardo.alonso@uc3m.es (E.A.); rvergaz@ing.uc3m.es (R.V.); cdios@ing.uc3m.es (C.D.)

**Keywords:** organic photodetector, bulk-heterojunction, visible light communication

## Abstract

A visible light communication (VLC) system using an organic bulk heterojunction photodetector (OPD) is presented. The system has been successfully proven indoors with an audio signal. The emitter consists of three commercial high-power white LEDs connected in parallel. The receiver is based on an organic photodetector having as active layer a blend of poly(3-hexylthiophene) (P3HT) and phenyl C61-butyric acid methyl ester (PCBM). The OPD is opto-electrically characterized, showing a responsivity of 0.18 A/W and a modulation response of 790 kHz at −6 V.

## 1. Introduction

Since the discovery of conducting polymers organic bulk heterojunction (BHJ) optoelectronic devices have become very popular during the last years [1], due to the advantages they offer over inorganic ones: flexibility, low weight, small size, low cost, simple fabrication techniques, and the possibility of developing components over large areas by means of roll to roll [2] or ink-jet printing [3]. These devices can be manufactured onto glass or plastic substrates which make them perfect candidates for future wearable electronics applications [4]. In the field of detectors, the blend based on P3HT:PCBM has been widely used as active layer for organic solar cells (OSCs), having achieved efficiencies up to 5% for standard structures [5]. In recent years this polymer:fullerene blend has attracted great interest for use as an active material in organic photodetectors in the visible region [6–13]. These devices show high external quantum efficiency (EQE) compared to other organic blends, up to 76% [6], high modulation bandwidths up to 1 MHz [9], good responsivity (R) of ∼0.25 (A/W) [6,10], good specific detectivity (D*) values of 7 × 10^12^ cm Hz^½^/W and noise equivalent power (NEP) values of 2.8 × 10^−14^ W/Hz [6]. These values are comparable to inorganic devices and make OPDs suitable for a wide variety of applications. In this context, many studies are focused on integrating OPDs in systems for communication and instrumentation applications [14–19] and wearable electronics [4].

Even though OPDs are beginning to be used in many communication applications, to our knowledge there is no evidence that they have been integrated into a VLC system, a rapidly growing research technology [20] that has drawn interest from both research and industrial communities, e.g., the Visible Light Communications Consortium [21] and the IEEE Task Group 802.15.7 [22]. VLC systems have many industrial applications areas such as visual signaling and communications, e.g., from a traffic signal to a car [23], communications using an information display [24], point to point communications between two peripherals [25], and positioning systems [26]. However the most popular applications of VLC systems are illumination and communication. The most straightforward advantage of using organic optoelectronic components is the possibility to integrate VLC receptors in wearable clothes.

VLC systems have developed greatly during the last few years as white LEDs started to replace light bulbs due to their higher efficiency, lower cost, and longer lifetimes. Standard white LEDs used for illumination can typically be modulated at high speeds (up to several MHz) so they can be used for illumination and for wireless data transmission simultaneously without significant cost. The IEEE consortium finished VLC system standardization in the 802.15.7 VLC Task Group [27]. Two types of white LEDs are used for illumination and communication applications: (i) separate red-green-blue emitters or (ii) blue emitter combined with a phosphor that emits in the yellow region. The latter approach is usually the preferred option because of its lower complexity. However, these devices have lower bandwidth due to the slow response of the phosphor, typically ≈3 MHz [24]. This problem can be solved using an optical filter, although this incurs a power penalty. Many authors have focused on optimizing these kinds of systems, trying to maximize the system transmission capacity using spectrally efficient modulation formats with digital signal processing [28] or employing wave division multiplexing (WDM) transmission [29].

In this work we demonstrate for the first time a real time VLC system using as receiver a BHJ organic photodetector based on P3HT:PCBM. The system has been successfully proven indoors with an audio signal. The paper is organized as follows: in Section 2.1 we fully describe the OPD fabrication and characterization set up, in Section 2.2 we detail the VLC system layout and in Section 3 we show the photodetector characterization and present the performance of the complete system.

## 2. Experimental Section

### 2.1. OPD Fabrication and Characterization Set up

**Figure 1.**
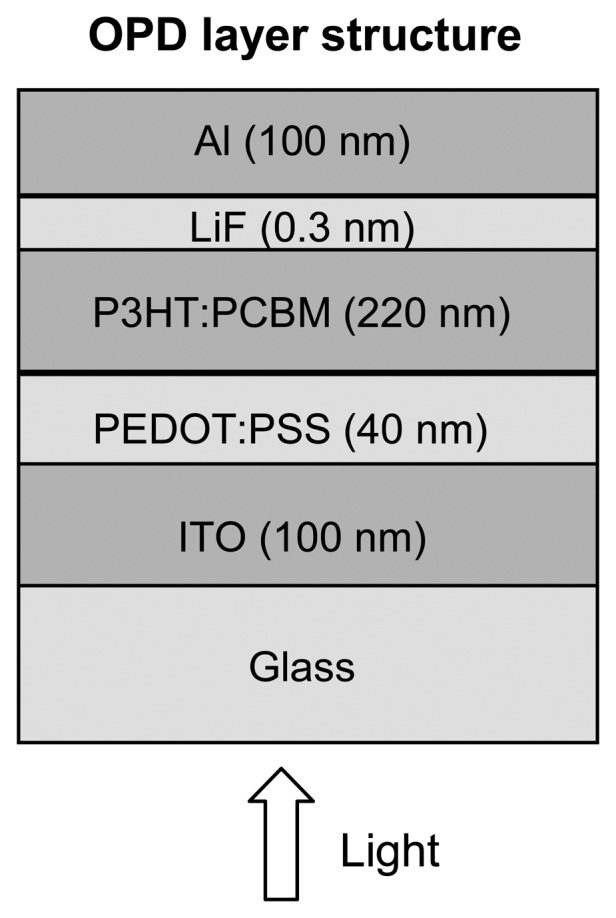
Schematic illustration of the OPD structure.

Commercial indium tin oxide coated glass substrates (thickness = 100 nm) were cleaned in ultrasonic baths using different solvents and exposed to UV-ozone for 20 min. The hole transport layer of poly(3,4-ethylenedioxythiophene)-poly(4-styrene sulfonate) (PEDOT:PSS) was spin coated at 3,000 r.p.m. and dried on a hot plate at 130 °C for 10 min, yielding a thickness of 40 nm. The active layer blend of P3HT:PCBM, in a 1:0.7 ratio in dichlorobenzene at 3% wt was spin coated at 800 r.p.m. yielding a thickness of 220 nm. Blends were annealed at 150 °C on a hot plate during 15 min. Finally, a thin layer of LiF (0.3 nm) and a layer of Al (100 nm) were thermally evaporated on top of the device with a vacuum pressure lower than 10^−5^ mbar. Devices were encapsulated with a glass tap and an UV-curing adhesive. The whole fabrication process was carried out into a glove-box with N_2_ atmosphere, except for the PEDOT:PSS coating. Device active area is 9 mm^2^. Figure 1 shows the device structure.

EQE and I-P characteristics were measured with a lock-in amplifier (SRS 830, Stanford Research Systems, Sunnyvale, CA, USA) and a UV808 UV-enhanced silicon detector (Newport, Irvine, CA, USA). EQE measurement also required a 100 W halogen tungsten lamp and a mechanical chopper Thorlabs (Dachau/Munich, Germany). I-V curves, I-P characteristics and frequency response were all performed illuminating the device with a TLLG5400 green LED ((λ = 530 nm, Vishay, Selb, Germany) since absorbance of P3HT:PCBM films presents a maximum at 510 nm [30,31]. I-V curves were generated with a semiconductor parameter analyzer Agilent 4155C and a source generator Agilent 41501B (both from Agilent Technologies, Santa Clara, CA, USA). The OPD frequency response was evaluated modulating the optical emission with a transconductance amplifier that varied the current flow through the LED. The detector under study was reverse biased and connected to an equivalent 50 Ω load. Frequency response has been characterized using a high sensitive lock-in amplifier for frequencies below 100 kHz and an electronic spectrum analyzer (ESA Agilent EXA N910A), suitable for frequencies above 60 kHz. Spectral measurement of the emission of the white LED was made with a USB2000 portable spectrometer (Ocean Optics, Dunedin, FL, USA).

### 2.2. VLC System Layout

**Figure 2.**
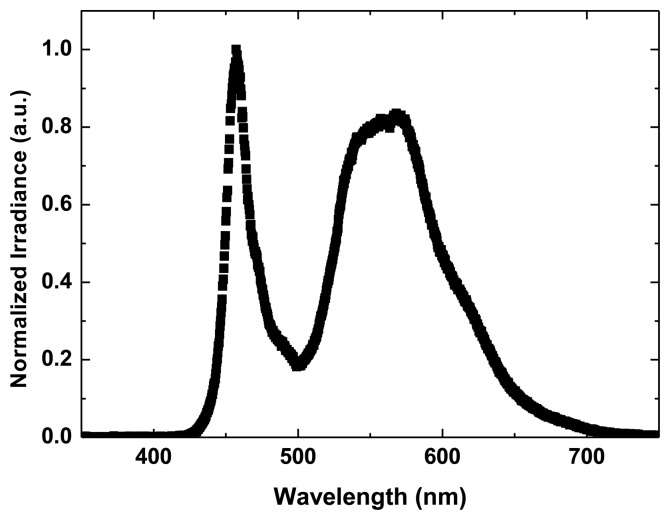
Emission spectrum of a W11492 white LED.

A VLC system consists of a transmitter, a propagation channel and a receiver. In our case, we have used as optical source three commercial W11492 high-power white LEDs from Seoul Semiconductor Co., Ltd. (Ansan-City, Korea), connected in parallel. Figure 2 depicts the emission spectrum of one of these LEDs, showing a peak at 457 nm and a broad phosphor spectrum at higher wavelengths. This type of optical source is chosen as it is considered standard and inexpensive. As reported elsewhere [32], the long decay time of the phosphor usually results in a limitation on the overall bandwidth available of the whole VLC system.

**Figure 3.**
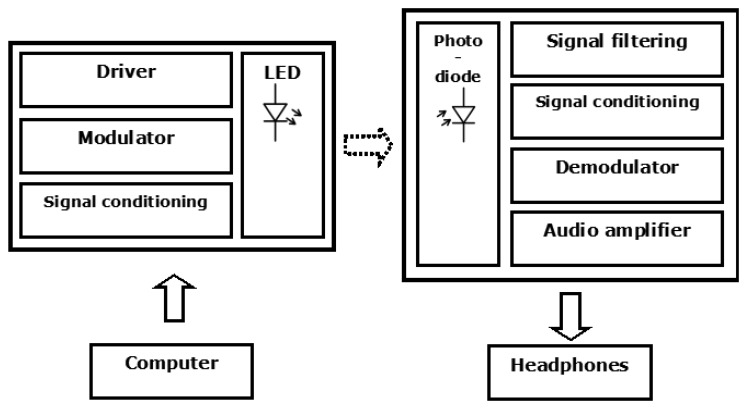
Block diagram of the VLC system.

We built a preliminary indoor visible light communication system prototype to demonstrate the ability to transmit high quality audio signals as well as other information at low-medium data rates. Figure 3 shows the block diagram of the designed VLC system. The system can operate at a maximum vertical distance of 65 cm. At the transmitter, we used three W11492 white LEDs in parallel under 700 mA average driving current and a forward voltage of 3.5 V. The rest of the electro-optical performance data of the LED can be found in its datasheet [33]. The LED was driven by On-Off Keying (OOK) modulated signals from a computer connected by an RS232 wire and its corresponding electric interface.

At the receiver, we use an organic bulk heterojunction photodetector that will be described in the next section. The receiver was pointed towards the transmitter in order to collect the maximum optical radiation coming from the LED. We have checked that other major noise sources do not influence this VLC system. The ambient light noise power induced by the scattered sunlight inside the office (with standard glass windows) and the diffused light from fluorescent lamps modulated with power line frequency 50 Hz have been removed electronically using a high-pass filter. Experimental measurements in the VLC system in presence of both noise sources have demonstrated a negligible influence of noise in the output signal after post-processing was performed. Therefore, no additional optical system such as bandpass filter has been coupled to the photodetector. Moreover, no concentrator was used to increase the detection area.

## 3. Results and Discussion

### 3.1. OPD Optoelectronic Performance

**Figure 4.**
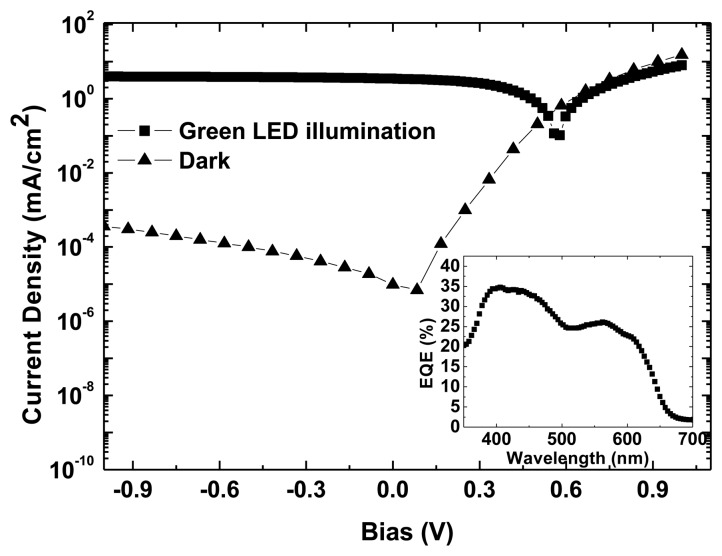
J-V characteristic of the OPD in dark (triangles) and under green- LED illumination (squares). Inset shows OPD external quantum efficiency at 0 V.

Figure 4 shows the current density *versus* voltage (J-V) curves of the photodetector in the dark and under green LED illumination. The photocurrent density under reverse bias remains in a quasi-constant range from 3.8 mA/cm^2^ at −1 V up to 4 mA/cm^2^ at −3 V, and the dark current at −1 V was 3.6 × 10^−4^ mA/cm^2^. Dark J-V curves present an asymmetric behavior with a good rectification ratio at ±1 V of 4 × 10^4^, suggesting an effective collection of photoinduced charge carriers, even at low reverse bias [34]. The figure inset shows the EQE measurement at zero bias voltage with a maximum value of 35% at 414 nm.

**Figure 5.**
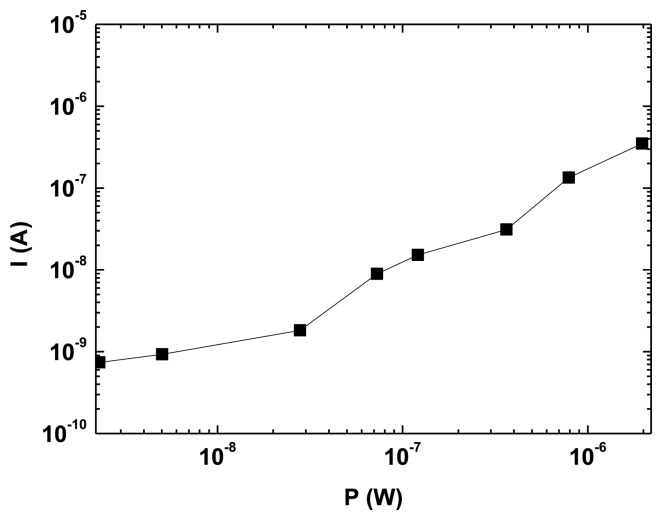
Photogenerated current *versus* incident optical power of a green LED at zero voltage bias.

Figure 5 shows the typical I-P characteristic of a photodetector, the photogenerated current *versus* the incident optical power. The responsivity, R (A/W), can be calculated from the experimental data obtaining values ranging from 0.17 A/W at 2 μW up to 0.18 A/W at 5 nW. R can also be estimated in terms of the wavelength of the incident light and quantum efficiency as:

(1) $$R=\frac{\eta e\lambda }{hc}$$

where e is the electron charge, λ is the wavelength, h is the Plank constant, c is the light velocity and η is the quantum efficiency. Taking the value of the EQE measurements η = 0.25 at 530 nm, the theoretical responsivity is found to be 0.11 A/W, agreeing reasonably well with the experiment.

Assuming that the shot noise current associated to the dark current is dominant over the thermal noise, the noise equivalent power (NEP, in W/Hz^1/2^) can be expressed as [35]:

(2) $$\text{NEP}=\frac{\sqrt{\langle I_{N}^{2}\rangle }}{R}=\frac{\sqrt{2eI_{D}}}{R}$$

where I_D_ is the OPD dark current. The NEP obtained for our photodetector is 9.5 × 10^−13^ W/Hz^1/2^. The specific detectivity (D*, Jones), that indicates the ability to detect low levels of incident power, is given by:

(3) $$D^{*}=\frac{\sqrt{A}}{\text{NEP}}$$

where A is device area. We calculate a D* of 3.15 × 10^11^ Jones. These values of NEP and D* are in the same order of magnitude to those obtained using the same structure and materials by other authors [10] and good enough for this VLC application.

**Figure 6.**
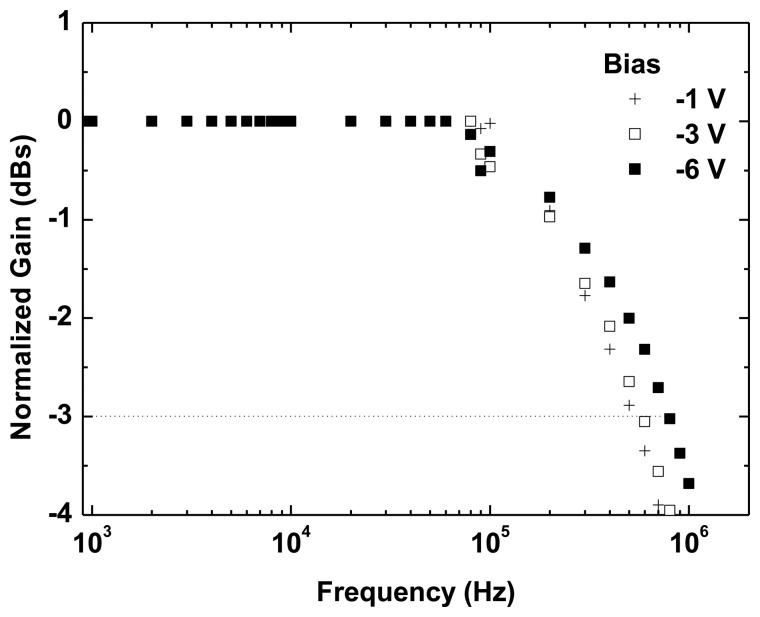
Modulation response of the OPD biased at −1 V, −3 V and −6 V under green-LED illumination. Frequency response was characterized with a 100-kHz lock-in amplifier setup and an electronic spectrum analyzer from 60 kHz upwards.

Figure 6 shows the normalized frequency response of the OPD at −1 V, −3 V and −6 V under green LED illumination. Measurements carried out with the lock-in amplifier (up to 100 kHz) are shown along with the characterization performed with the high frequency ESA (from 60 kHz upwards). The photodetector bandwidth increases with reverse bias reaching up to 790 kHz at −6 V. Further increasing bias voltage does not significantly improve the −3 dB cutoff frequency. These values are higher than that measured for similar organic standard BHJ photodetectors [6,7] and very close to the state-of-the-art [9]. This OPD speed is high enough for many communication and instrumentation applications.

### 3.2. Integration of the OPD in a VLC System

The implemented VLC system was used to transmit an audio signal generated from a computer. Data was recorded setting two distances between the optical emitter and the photodetector, 20 cm and 40 cm. We have measured the illuminance incident on our OPD, obtaining 300 lux at 40 cm and 1,150 lux at 20 cm. This leads to a decrease from 1.7 to 0.4 W/m^2^ at 555 nm. In both situations, the OPD is efficiently enough to generate the necessary current to recover the transmitted signal, according to Figure 5. For this specific application, the system can operate at a data rate lower than the modulation bandwidth of the white LED used, thus, simple driving schemes are considered. Data-rate is 50 Mb/s for Non-Return to Zero (NRZ). On-Off Keying has been used for transmitting the audio signal and the analog audio signal has been retrieved in order to validate the system.

**Figure 7.**
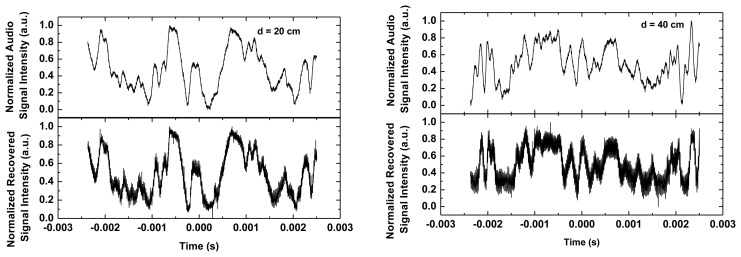
Acquisition made by a digital oscilloscope of the audio signal transmitted and the signal recovered in the receiver at two different distances, 20 cm and 40 cm. The retrieved signal exhibits a residual carrier noise at 50 kHz of 48 mV_rms_, easily removable by improving the filtering or by slightly increasing the carrier frequency.

Figure 7 depicts the audio signal generated from the computer and transmitted by the emitter, and the signal recovered at the receiver. The figure shows a good reconstruction of the emitted signal at the organic receiver. A small delay can be observed due to the electronic post-processing of the output signal coming from the photodetector. The bandwidth of the photodetector for these bias conditions is around 500 kHz, meaning that the OPD response has no influence in such a delay. However, the feedback loop of the transimpedance amplifier used as signal first conditioning is around 100 kHz, and the measured delay is around 10 μs. A simple first-order analogue equalizer was used in the receiver producing substantial improvement in data-rates. Probably, more complex electronic approaches would lead to higher data rates [36].

In the Supplementary Information authors have included a multimedia video file showing the performance of the whole system. It can be observed that when an object is placed between the emitter and the receiver, the communication is interrupted.

## 4. Conclusions

In this work we have demonstrated a visible light communication system using an organic bulk heterojunction photodetector for receiving an audio signal. This is, to our knowledge, the first time that an organic photodiode based on P3HT:PCBM has been implemented within a VLC system. The complete communication system shows good performance for an audio signal generated from a computer. The OPD bandwidth is characterized to be 790 kHz, suggesting that it could be employed in communication applications requiring higher data rate.
